# CD69-oxLDL ligand engagement induces Programmed Cell Death 1 (PD-1) expression in human CD4 + T lymphocytes

**DOI:** 10.1007/s00018-022-04481-1

**Published:** 2022-08-05

**Authors:** María Jiménez-Fernández, Cristina Rodríguez-Sinovas, Laia Cañes, Carme Ballester-Servera, Alicia Vara, Silvia Requena, Hortensia de la Fuente, José Martínez-González, Francisco Sánchez-Madrid

**Affiliations:** 1 Hospital Universitario de la Princesa, Universidad Autónoma de Madrid (UAM), Instituto de Investigación Sanitaria del Hospital Universitario de La Princesa (IIS-IP), c/ Diego de León, 62, 28006 Madrid, Spain; 2 Centro Nacional de Investigaciones Cardiovasculares (CNIC), Madrid, Spain; 3 Institut de Recerca Hospital de la Santa Creu i Sant Pau (IRHSCSP), IIB-Sant Pau, Barcelona, Spain; 4 CIBER de Enfermedades Cardiovasculares (CIBERCV), Madrid, Spain; 5 Instituto de Investigaciones Biomédicas de Barcelona - Consejo Superior de Investigaciones Científicas (IIBB-CSIC), IIB-Sant Pau, C/ Rosselló, 161, 08036 Barcelona, Spain

**Keywords:** CD69 leucocyte activation receptor, oxLDL, Cardiovascular disease, Programmed Death 1 (PD-1), Anti-inflammatory response, Inflamed aorta

## Abstract

The mechanisms that control the inflammatory–immune response play a key role in tissue remodelling in cardiovascular diseases. T cell activation receptor CD69 binds to oxidized low-density lipoprotein (oxLDL), inducing the expression of anti-inflammatory NR4A nuclear receptors and modulating inflammation in atherosclerosis. To understand the downstream T cell responses triggered by the CD69-oxLDL binding, we incubated CD69-expressing Jurkat T cells with oxLDL. RNA sequencing revealed a differential gene expression profile dependent on the presence of CD69 and the degree of LDL oxidation. CD69-oxLDL binding induced the expression of NR4A receptors (NR4A1 and NR4A3), but also of PD-1. These results were confirmed using oxLDL and a monoclonal antibody against CD69 in CD69-expressing Jurkat and primary CD4 + lymphocytes. CD69-mediated induction of PD-1 and NR4A3 was dependent on NFAT activation. Silencing NR4A3 slightly increased PD-1 levels, suggesting a potential regulation of PD-1 by this receptor. Moreover, expression of PD-1, CD69 and NR4A3 was increased in human arteries with chronic inflammation compared to healthy controls, with a strong correlation between *PD-1* and *CD69* mRNA expression (*r* = 0.655 *P* < 0.0001). Moreover, PD-1 was expressed in areas enriched in CD3 infiltrating T cells. Our results underscore a novel mechanism of PD-1 induction independent of TCR signalling that might contribute to the role of CD69 in the modulation of inflammation and vascular remodelling in cardiovascular diseases.

**Supplementary Information:**

The online version contains supplementary material available at 10.1007/s00018-022-04481-1.

## Introduction

The early lymphocyte activation antigen CD69 is a type II C-lectin membrane receptor readily induced upon leukocyte stimulation. Initial in vitro studies of CD69 suggested a pro-inflammatory role for this molecule [1]. However, growing evidence from in vivo analyses in mice lacking CD69 highlighted its role as a molecular brake to control exacerbated inflammatory responses [2]. Animal models including collagen-induced arthritis, autoimmune experimental myocarditis, allergic asthma and colitis, among others, clearly established that CD69 exerts an immune regulatory and protective role in inflammation [2–4]. CD69 acts through different mechanisms, mainly inhibiting Th17 cell differentiation and regulating the Th17/Treg cell balance. Hence, expansion of Th17 cell activity was observed in the absence of CD69; conversely, the suppressive function of CD4 + CD25 + FoxP3 + Tregs was diminished in CD69-deficient mice, revealing the importance of CD69 expression on this T cell subset [5]. The mechanisms by which CD69 controls Treg activity include upregulation of TGF-β [4, 6], elevated production of the cytokine IL-10 and expression of GITR, CD38, ICOS and CTLA-4 [7]. CD69 is required for the expansion of Tregs in the thymus (tTregs) through the regulation of BIC/microRNA 155 and its target, suppressor of cytokine signalling 1 (SOCS-1) [8].

Understanding the pathophysiologic function of CD69 has been challenging by the absence of known ligands. Recently, CD69 has been identified as an oxidized low-density lipoprotein (oxLDL) receptor in human CD4 + T lymphocytes [9]. OxLDL-CD69 binding triggers a protective effect, enhancing the anti-inflammatory transcription factors NR4A1 and NR4A3 (NOR-1), downregulating proinflammatory cytokines and promoting Treg differentiation, thereby modulating atherosclerosis [9].

An increasingly number of immunoregulatory molecules such as CTLA-4, PD-1, ICOS, LAG-3 and TIGIT inhibit the activity and proliferation of T lymphocytes [10, 11]. The inhibitory receptor programmed cell death-1 (PD-1, CD279) is a key therapeutic target to treat immune and inflammatory disorders as well as to stimulate the anti-tumour immune response [12]. PD-1 and its ligands PD-L1 (B7-H1, CD274) and PD-L2 (B7-DC, CD273) belong to the B7-CD28 family and are highly regulated during normal immune responses [13].

Here, we report that the binding of oxLDL to CD69 induces the expression of PD-1 through the activation of nuclear factor of activated T cells 1 (NFAT) in human T cells. Analysis of human inflammatory artery samples showed a high expression of CD69 and PD-1 and strong correlation between them. Our data demonstrate that PD-1 upregulation is a downstream mechanism involved in the negative regulation of the inflammatory response upon CD69–ligand engagement.

## Materials and methods

### Cell cultures

Human T lymphocyte Jurkat cell line stably transfected or not with CD69 (JKCD69 and JKwt, respectively) was used [9]. The cells were maintained with RPMI 1640 + GlutaMAX™ (*Gibco*) supplemented with 10% of heat-inactivated foetal bovine serum (FBS) (*GE Healthcare Hyclone,*), 0.1 U/ml penicillin and streptomycin (*Lonza*) and the selection antibiotic geneticin G418 (*InvivoGen*) at 0.5 mg/ml. Both cell lines were incubated at 1·10^6^ cells/ml with lipoproteins in their native or oxidized form at a concentration of 50 µg/ml in 2% FBS medium the time indicated in each experiment. Peripheral blood mononuclear cells were purified by density gradient with Pancoll human (*PAN-Biotech*) from healthy donors and CD4+ T lymphocytes isolated using the EasySep™ Human CD4+ T Cell Isolation Kit (*Stemcell)*.

### Low-density lipoproteins (LDL) isolation and oxidation.

Native LDL (nLDL) were isolated from pooled plasma of healthy blood donors of the Barcelona area [14]. The procedure was approved by the Ethics Committee of the Hospital de la Santa Creu i Sant Pau (Barcelona, Spain) and was conducted in accordance with the Declaration of Helsinki. Briefly, pooled plasma was centrifuged (80,000×*g* for 30 min at 4 °C) to remove chylomicrons. LDL (*d* = 1.019–1.063 g/mL) were isolated by potassium bromide density gradient ultracentifugation using a Beckman Coulter Optima™ L-100 XP ultracentrifuge and a Beckman 50.2 Ti rotor (Beckman Coulter) at 36,000 rpm for 18 h at 4 °C (gmax = 156,000). The LDL fraction was dialysed four times against 200 volumes of PBS for 24 h. All solutions were deoxygenated by N_2_ bubbling. LDL were sterilized by filtration through a low-protein-binding non-pyrogenic filter (Millex-GV, Millipore). Oxidized LDL (oxLDL) with different degrees of oxidation were prepared by exposing nLDL to 10 µM CuSO_4_ at 37 °C for increasing times. LDL were stored at 4 °C and protected from exposure to light. The content of protein (BCA protein assay™, Pierce) and cholesterol (Cholesterol Assay Kit™, Abcam) was determined by colorimetric assays. LDL were free from contamination from other lipoproteins (as determined by electrophoresis) or endotoxin (as determined by a Limulus assay) (Chromogenic Endotoxin Quan kit™, Pierce). Thiobarbituric acid-reactive substances (TBARS) content of LDL was used as an indirect evaluation of lipid peroxidation [14]. In the present study, we used nLDL (TBARS below 1 nmol malonaldehyde (MDA)/mg of LDL protein), moderately oxidized LDL (moxLDL; TBARS around 10 nmol MDA/mg of LDL protein) and highly oxidized LDL (hoxLDL; TBARS between 25 and 50 nmol MDA/mg of LDL protein).

### mRNA extraction and RNA-seq analysis

For experiments aimed to analyse mRNA levels, cells were incubated for 45 min, 4 and 8 h under the following conditions: without LDL, with nLDL or with oxLDL. Then, the cells were collected, rinsed once with phosphate-buffered saline (PBS) and preserved in QIAzol Lysis Reagent (*Qiagen*) at  − 80 °C until they were processed. Total RNA was isolated using the miRNeasy Mini Kit (*Qiagen*), and DNase digestion was performed as recommended. The cDNA was obtained by the GoScript™ Reverse Transcriptase Kit (*Promega*) according to the manufacturer’s protocol.

For the RNA-sequencing, JKwt and JKCD69 cells were incubated for 4 h with nLDL moxLDL and hoxLDL as an exploratory study. RNA was quantified by spectrophotometry (ND-1000; NanoDrop Technologies), and RNA quality was determined by microcapillary electrophoresis on an Agilent 2001 Bioanalyzer (Agilent Technologies) with RNA 6000 Nanochips. The samples were sequenced and processed by the Bioinformatics Unit and the Genomics Unit from the Centro Nacional de Investigaciones Cardiovasculares Carlos III (CNIC), Madrid, Spain. 200 ng of total RNA was used to generate barcoded RNA-seq libraries using the NEBNext Ultra II Directional RNA Library Prep Kit (New England Biolabs). Briefly, poly-A + RNA was purified using poly-T oligo-attached magnetic beads followed by fragmentation and then first and second cDNA strand synthesis. Second strand was synthesized with U ribonucleotide instead of T. Next, cDNA 3′ ends were adenylated and the adapters were ligated, second strand were fragmented by USER enzyme that cut the uracils to preserve the directionality of the original RNA. Then the libraries were amplified by PCR. Directional RNA libraries were determined using the Agilent 2100 Bioanalyzer high-sensitivity DNA chip. Libraries were sequenced, Single-End 1 × 60, on a HiSeq 2500 (Illumina) and processed with RTA v1.18.66.3. FastQ files for each sample were obtained using bcl2fastq v2.20.0.422 software (Illumina). FastQ files were pre-processed by FastQC (Babraham Bioinformatics—FastQC A Quality Control tool for High Throughput, available online at: https://www.bioinformatics.babraham.ac.uk/projects/fastqc/), to assess reads quality and Cutadapt to trim sequencing reads [15]. Resulting reads were mapped against reference transcriptome GRCh38.91 and quantified using RSEM [16]. Expected expression counts calculated with RSEM were then processed with an analysis pipeline that used the Bioconductor package EdgeR for normalization (using TMM method) and expression ratio calculation, taking only into account those genes expressed with at least 1 count per million (CPM) in at least one sample [17]. The gene expression was normalized of every sample relative to its control for each cell line (LDL vs. untreated) and then compared the changes detected with increasing LDL oxidation degrees in JKCD69 respect to JKwt. As an exploratory study, genes were selected as follows. On the one hand, genes candidates were selected using a threshold of fold change > 1.8 in JKCD69 cells treated with hoxLDL. Heatmap and clustering for those genes were accomplished using Galaxy web platform and the public server at usegalaxy.org. Original values were Log (*x* + 1)-transformed, columns were clustered using Euclidean method and scaling the data by row. Principal component analysis (PCA) was performed using Minitab Statistical Software (Minitab, LLC, Pennsylvania, USA). On the other hand, the gene expression of selected genes was compared in JKCD69 incubated with moxLDL and hoxLDL to the control samples (JKwt samples and JKCD69 resting and nLDL).

### Quantification of gene expression

Total RNA from human tissues was isolated using TRIsure™ reagent (Bioline) and reverse transcribed into cDNA using the high-capacity cDNA Reverse Transcription Kit (Applied Biosystems). Gene expression for *NR4A* receptors in human samples and cell culture studies was evaluated by real-time PCR using the SensiFAST probe Hi-ROX mix (Bioline), the ABI PRISM 7900HT sequence detection system (Applied Biosystems) and specific Taqman probes (*Applied Biosystems*) for *NR4A1*, (Hs00172437_m1) and *NR4A3* (Hs00175077_m1). Alternatively, mRNA levels were assessed with the GoTaq^®^ qPCR Master Mix system (*Promega*) for SYBR Green real-time PCR analysis. The specific primers pairs (*PD-1* forward CTC CAG GCA TGC AGA TCC, reverse GGC CTG TCT GGG GAG TCT A; *CD69* forward CAA GTT CCT GTC CTG TGT GC and reverse GAG AAT GTG TAT TGG CCT GGA; and *GAPDH* forward AGC CAC ATC GCT CAG ACA C and reverse GCC CAA TAC GAC CAA ATC C) were designed using the Roche Life Science web (Penzberg, Germany) and synthesized by Metabion (Steinkirchen, Baviera, Germany). Detection of amplified products was performed with the CFX384 Touch™ Real-Time PCR detection system (*Bio-Rad*). Data were analysed with the 3.1 version of the CFX Manager software (*Bio-Rad*). The PCR protocol consisted of a first denaturation step of 10 min at 95 °C followed by 40 cycles of 10 s at 95 °C and at 60 s of annealing/extension step at 60 °C and a 10 s at 95 °C before the melting curve was achieved. The real-time qPCRs were performed in triplicate for all the targets. The relative mRNA levels were determined using the comparative threshold (CT) method after checking primers efficiency. Normalization was accomplished to *β-ACTIN* (Hs99999903_m1; for human tissue samples) or *GAPDH* levels. The relative mRNA levels were expressed as folds of change over untreated samples.

### Anti-CD69 antibody assays

Monoclonal antibody TP1/55 anti-CD69 employed was generated at the Hospital Universitario La Princesa (Madrid, Spain) as it has been previously described [1]. The JKwt and JKCD69 cells were cultured in supplemented medium at 1·10^6^ cells/ml with the antibody (10 μg/ml) and the crosslinker AffiniPure Fab Fragment Goat Anti-Mouse IgG, Fcγ Subclass 2b Specific (20 μg/ml, *Jackson ImmunoResearch*) for three days. As isotype control, the mouse IgG2b kappa isotype control antibody (10 μg/ml, *Thermo Fisher Scientific/Life*) was engaged and crosslinker was also added to this condition. For human primary CD4+ T cells experiments, cells were activated with phorbol 12-myristate 13-acetate (PMA) (50 ng/ml, *Sigma-Aldrich*) and ionomycin (Io) (1 μg/ml, *Sigma-Aldrich*) for 3 h to induce the CD69 expression prior the addition of anti-CD69 monoclonal antibody and crosslinker as mentioned before.

### Analysis of protein surface expression by flow cytometry

The protein levels were measured after different time points of incubation by flow cytometry in a BD FACS Canto II Flow Cytometer and analysed with FlowJo software (FlowJo, LLC). Cells were rinsed twice with PBS (1X) and resuspended with PBS and Ghost Dye Red 780 (1:1000 dilution, *Tonbo Biosciences*) for 30 min on ice. After that, cells were rinsed once with PBS-0.2% Bovine Serum Albumin (BSA) (*PanReac AppliChem*) and resuspended in 50 μl of this buffer with the PE anti-human CD279 (PD-1) (EH12.2H7, 1:100 dilution, *BioLegend*) antibody for 30 min on ice. The analysis strategy was to compare the mean fluorescence intensity between treated and untreated live cells for oxLDL experiments. For anti-CD69 monoclonal antibody engagement experiments, samples were incubated with mouse serum (1:100 dilution, *Sigma-Aldrich*) for 15 min before adding the same volume with flow cytometry antibodies. Percentage of positive cells for PD-1 were represented at different times.

### NR4A3 interfering by siRNA and cyclosporine A assays

Gene knockdown of *NR4A3* was performed using ON-TARGETplus SMARTpool small interfering RNA (siRNA) and ON-TARGETplus Control Pool Non-targeting Pool as scramble (*Horizon*) by electroporation. Cells were washed once with Hanks’ Buffered Saline Solution (HBSS, *Lonza*) and once with Opti-MEM reduced serum medium (*Gibco*) before electroporate. After 48 h, specific knockdown was confirmed by RT-qPCR of mRNA levels and experiments were performed. Cyclosporine A (CsA) (5 μg/ml, SML1018 from *Sigma-Aldrich,* Burlington, MA, USA), PMA (50 ng/ml, *Sigma-Aldrich*) and Io (1 μg/ml, *Sigma-Aldrich*) were employed when indicated.

### Human artery sampling and preservation

Abdominal aortic samples were collected from patients undergoing open repair surgery for abdominal aortic aneurysm at the Hospital de la Santa Creu i Sant Pau (Barcelona, Spain) and from multi-organ donors [18]. The study was approved by the Ethics Committee of the Hospital de la Santa Creu i Sant Pau (Barcelona, Spain) and was conducted in accordance with the Declaration of Helsinki. Samples were split and processed for conventional histological staining/immunohistochemistry or frozen in liquid nitrogen and stored at − 80 °C for RNA and protein extraction. The specimens for conventional staining/immunohistochemistry were immersed in fixative solution (4% paraformaldehyde/0.1 M phosphate-buffered saline, pH 7.4). After overnight treatment, they were sectioned into blocks and embedded in paraffin. Vessels were cut into 4-µm-thick sections that were mounted on coated glass microscope slides (FLEX IHC, *Dako*, Santa Clara, CA, USA) and stored at room temperature until tested. The presence of inflammation in the aortic samples from patients selected for this study was confirmed by hematoxylin and eosin staining. Healthy control aortas from multi-organ donors were macroscopically normal and devoid of inflammatory lesions as determined by hematoxylin and eosin staining.

### Western blot analysis

Human specimen samples for western blotting were snap-frozen in liquid nitrogen and stored at -80ºC. Human protein extracts were obtained using ice-cold lysis buffer as described [19]. Protein content of lysates was determined by the bicinchoninic acid (BCA) protein assay (Pierce) using bovine serum albumin as standard. Protein lysates (15 µg/lane) were resolved using sodium dodecyl sulphate–polyacrilamide gel electrophoresis (SDS-PAGE) and transferred to polyvinylidene difluoride membranes (*Immobilon*, *Merck-Millipore*; Burlington MA, USA, IPVH00010). Blots were blocked with 5% non-fat dry milk in TBST buffer (100 mM sodium chloride, 10 mM Tris–HCl pH 7.5, 0.05% Tween-20) at room temperature for 1 h. Membranes were then incubated overnight with mouse monoclonal antibodies against: PD-1 (ab52587, *Abcam*, Cambridge, UK), CD69 (sc-373799, *Santa Cruz Biotechnology*, Inc., Dallas, TX, USA), NOR-1 (H00008013-M06, *Abnova*, Taipei, Taiwan) or CD3 (M7254, *Dako*). Appropriate horseradish peroxidase-conjugated secondary antibodies (*Dako*) and the Luminata™ Western HRP Substrate (*Immobilon*, *Merck-Millipore*) were used to detect bound antibodies. The size of detected proteins was estimated using protein molecular mass standards (Hyperpage Prestained Protein Marker; *Bioline*, Paris, France). ß-actin (A5441, *Sigma-Aldrich*) was used to verify equal loading of protein on each lane.

## Immunohistochemical analysis

Tissue sections (5 µm thick) were deparaffinised in xylene, rehydrated in graded ethanol solutions and subjected to heat-induced antigen retrieval either in 10 mM citrate buffer at pH 6 (for CD69 and NOR-1 detection) or in Tris–EDTA buffer pH 9.0 (for PD-1 detection). Then, slides were treated with 3% hydrogen peroxide in methanol for 30 min to block endogenous peroxidase activity. Subsequently, slides were incubated with 10% normal serum and endogenous avidin and biotin blocked using a commercial kit (*Vector Laboratories*, Burlingame, CA, USA). Then samples were incubated overnight at 4 °C with the following primary antibodies against: PD-1 (ab237728, *Abcam*) CD69 (sc-373799, *Santa Cruz Biotechnology*, Inc.) or NOR-1(H00008013-M06, *Abnova*). Samples were washed and then incubated with the appropriate biotinylated secondary antibodies for 1 h (*Vector Laboratories*, Burlingame, CA, USA). After washing three times in PBS, the avidin–biotin peroxidase complex (ABC, *Vector Laboratories*) was added for 30 min. Colour was developed by incubation with 3,3′-diaminobenzidine (DAB), and then, sections were counterstained with haematoxylin, dehydrated, cleared and mounted. Negative controls, in which the primary antibody was omitted, were included to test for non-specific binding of the secondary antibody.

### Statistical analyses

Data were analysed with GraphPad Prism 7.04 (GraphPad Software, San Diego, CA, USA). Significance differences were determined by two-way ANOVA when both cell lines were used with different treatments and one-way ANOVA to compare more than two means with one cell line. Tukey or Bonferroni post hoc tests were used to perform multiple comparisons as indicated. To compare two means in interfering experiments, unpaired *t* test and paired *t* test were used when appropriate. Differences from human samples experiments were analysed with Mann–Whitney test and Pearson product–moment correlation was applied. Differences were considered significant at *P* < 0.05. In all cases number of samples indicated at figure legends correspond to independent experiments.

## Results

### OxLDL-CD69 binding induces a differential transcriptional response in human CD4 + T lymphocytes

**Fig. 1:**
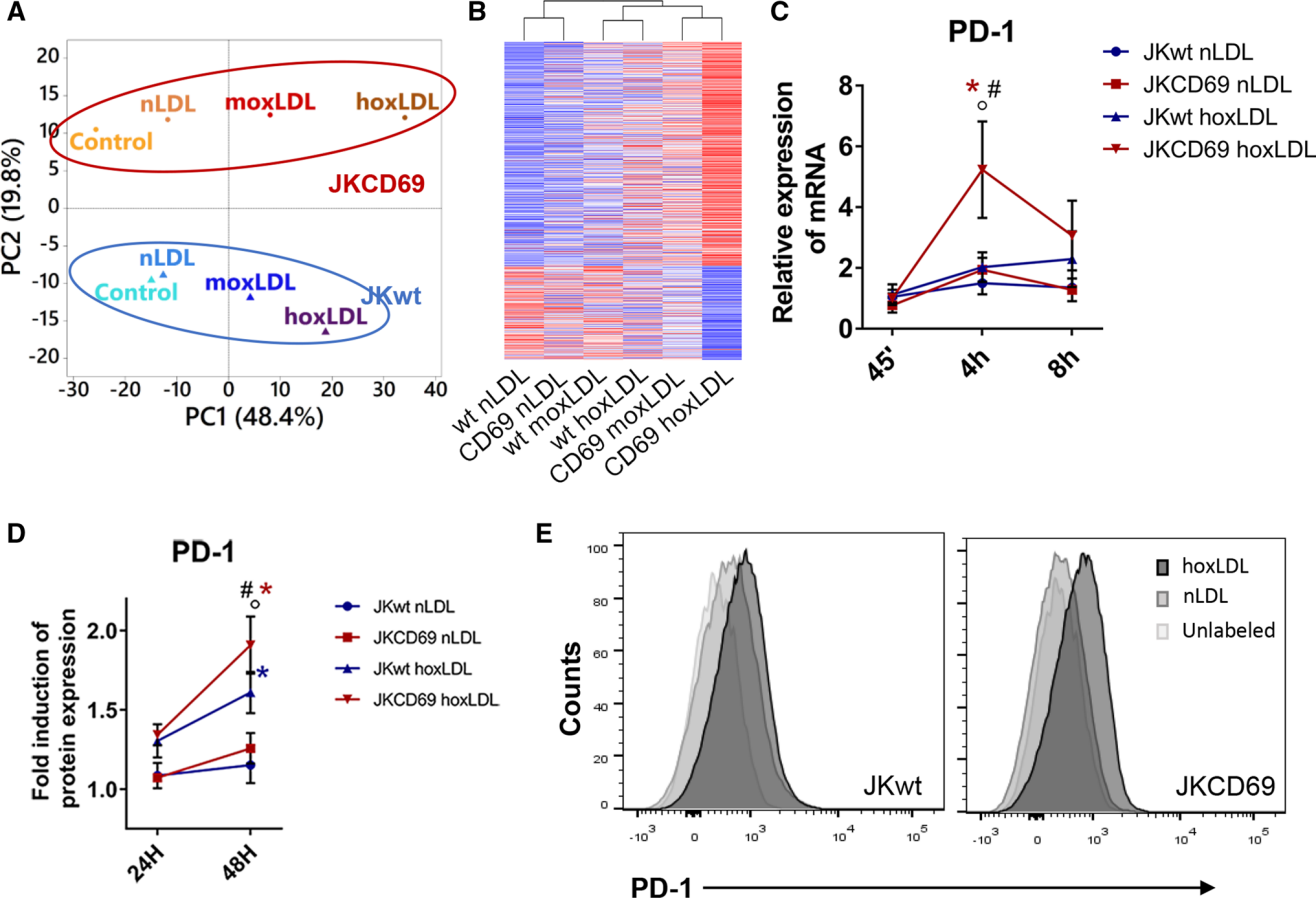
OxLDL-CD69 binding induces a differential transcriptional response increasing the PD-1 expression. **A** Principal component analysis (PCA) performed using the up- and downregulated genes from the RNA-Seq data of each incubation condition. The proportion of variability explained by each principal component (PC1 and PC2) are included in the axis. Blue samples correspond to JKwt and red samples to JKCD69. (nLDL: native LDL; moxLDL: moderately oxidized LDL; hoxLDL. highly oxidized LDL). **B** The oxidation degree of oxLDL induces a differential transcriptomic response. Heatmap based on the fold change of 577 upregulated and 242 downregulated genes in JKCD69 with the higher level of oxLDL. Data are shown as Ln (*x* + 1) and are clustered using correlation distance and average linkage. Up- and downregulated genes are shown in red and blue respectively. **C** Relative expression of PD-1 mRNA in JKCD69 and JKwt treated with hoxLDL. Data are mean ± SD (*n* = 3) and were analysed with two-way ANOVA (Tukey post hoc test). *Indicates significant differences (*P* < 0.05) with the treatment for each cell line (native vs. oxLDL), # JKCD69 vs. JKwt in the same condition, ◦ significant differences respect the previous time (45’ vs. 4 h, 4 h vs. 8 h). **D** Mean fluorescence intensity of PD-1 in both cell lines at different time points. Data are mean ± SD (*n* = 3) and were analysed with two-way ANOVA (Bonferroni post hoc test). *Indicates significant differences (*P* < 0.05) in the treatment for each cell line (native vs oxLDL), # JKCD69 vs. JKwt in the same condition, ◦ significant differences respect the time. **E** Histograms of a representative experiment of the induction of PD-1 in JKCD69 and JKwt after 48 h incubated with LDLs

To identify genes differentially expressed in response to the binding of oxLDL to CD69, JKCD69 and JKwt cells were incubated in the presence or absence of nLDL, and copper-oxidized LDL at two different degrees of oxidation, moxLDL and hoxLDL. To focus in those genes modified by hoxLDL in JKCD69 T cells, we used a cut-off of fold change > 1.8 and we identified 577 upregulated and 238 downregulated genes in this condition. Principal component analysis of these genes in all of the samples was represented (Fig. 1A). Statistical analysis showed clear differences in gene expression profile depending on the degree of LDL oxidation and the presence or absence of CD69 (Fig. 1A). Native LDL-treated cells exhibited similar expression profiles to those of untreated cells (Fig. 1A). The differences displayed in the PCA analysis are reflected on the heatmap clustering (Fig. 1B). Heatmap suggests that JKCD69 cells incubated with hoxLDL and moxLDL displayed a differential behaviour compared to JKwt with the same LDLs (Fig. 1B). Interestingly, we identified *PD-1* as one of the genes with higher levels of induction in JKCD69 *vs.* JKwt in response to oxLDL (Table 1; Supplementary Data 1). As an internal control, we identified increased expression of NR4A receptors, especially *NR4A3*, in JKCD69 treated with hoxLDL compared to JKwt treated with hoxLDL or JKCD69 cells treated or not with nLDL (Table 1).

**Table 1:** Potential genes of interest differentially expressed in JKCD69 vs. JKwt in response to the CD69-oxLDL binding

Official gene symbol	Fold change
*PDCD1*	4.91
*ENTPD1*	3.33
*KCNMB1*	3.09
*ENPP1*	2.64
*NR4A3*	2.41
*TLR6*	1.93
*NR4A1*	1.82
*PDGFC*	0.65
*TNFRSF4*	0.64
*RETN*	0.63
*IL26*	0.51
*CCR8*	0.35

### OxLDL-CD69 binding increases PD-1 expression

To confirm that oxLDL binding to CD69 upregulates PD-1 in T cells, the expression of *PD-1* was assessed by real-time PCR (RT-PCR) in JKCD69 and JKwt cells treated with hoxLDL. *PD-1* mRNA levels were significantly increased in JKCD69 after 4 h of incubation with oxLDL compared with JKwt cells, while nLDL did not induce *PD-1* expression in either cell line (Fig. 1C). Flow cytometry analysis showed that oxLDL increased PD-1 surface expression in JKCD69 compared to JKwt at 48 h, while no significant changes were elicited by nLDL (Fig. 1D, E). Jurkat cells express very low or negligible levels of CD69 (Supplementary Fig. 1) which is variable and dependent on the culture conditions and this could explain the low levels of PD-1 induction (Fig. 1D).

**Fig. 2:**
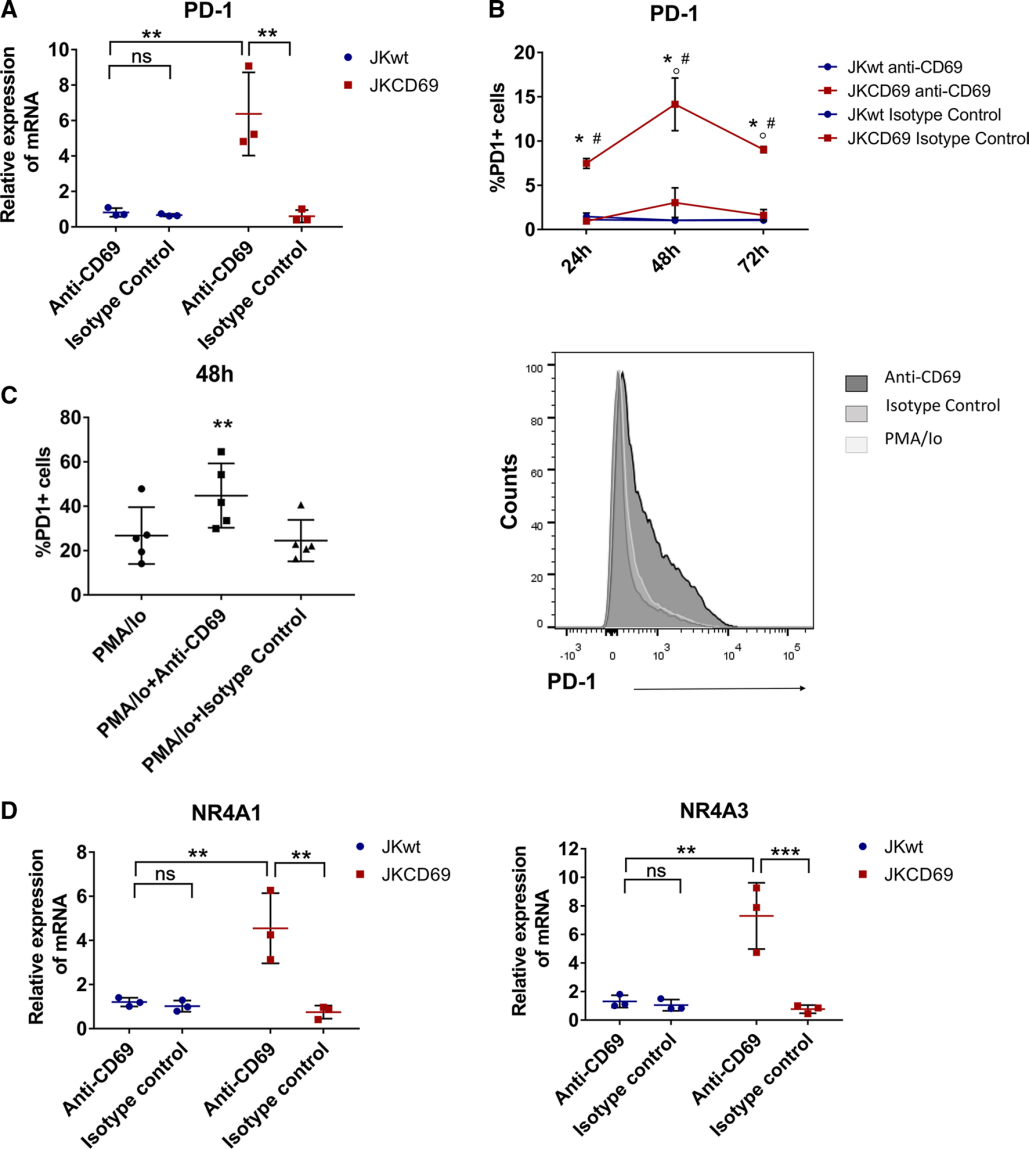
CD69 engagement induces the PD-1 mRNA and protein expression. **A** Relative expression of PD-1 mRNA induced by anti-CD69 TP1/55 antibody in JKCD69 after 4 h of stimulation. JKwt cells response was assessed as a control. An isotype control antibody was also used. Data are mean ± SD (*n* = 3) and **P < 0.01 differences were determined with two-way ANOVA (Tukey post hoc test). **B** Percentage of positive cells for PD-1 in both cell lines at different time points after engagement of the CD69 antibody TP1/55. Data are mean ± SD (*n* = 3), and differences were analysed with two-way ANOVA (Tukey post hoc test). *Indicates significant differences (*P* < 0.05) with the treatment for each cell line (antibody *vs*. isotype control), # JKCD69 *vs*. JKwt in the same condition, ◦ significant differences respect the previous time (24 h vs. 48 h, 48 h vs. 72 h). **C** Left, percentage of primary human CD4 + T cells expressing PD-1 after 48 h of stimulation. Data are mean ± SD (*n* = 5), and differences were determined with one-way ANOVA (Tukey post hoc test). **P < 0.01. Right, histogram of a representative experiment after 48 h of treatment. **D** Relative expression of NR4A1 and NR4A3 mRNA induced by anti-CD69 TP1/55 antibody in JKCD69 after 4 h. Data are mean ± SD (*n* = 3), and differences were analysed with two-way ANOVA (Bonferroni post hoc test). **P < 0.01. ***P < 0.001

To evaluate the specificity of CD69, we assessed whether the engagement of CD69 with specific antibodies triggers a similar response than oxLDL. JKwt and JKCD69 cells were cultured in the presence or absence of a CD69 antibody for 4 h, and mRNA levels were analysed by RT-PCR. In JKCD69 cells, the antibody significantly increased *PD-1* mRNA levels at 4 h (Fig. 2A). It also increased the amount of PD-1 detected on the cell membrane, with a peak expression at 48 h (Fig. 2B), similar to the induction effect observed with oxLDL. Moreover, the addition of anti-CD69 antibody after 3 h of stimulation with PMA/Io increased PD-1 expression in primary human CD4 + T cells from healthy donors (Fig. 2C). The CD69 antibody also induced the expression of *NR4A1* and specially *NR4A3* (Fig. 2D). No significant effects on gene expression were observed in T cells devoid of CD69 (JKwt).

### PD-1 induction through CD69 engagement is regulated by NFAT

**Fig. 3:**
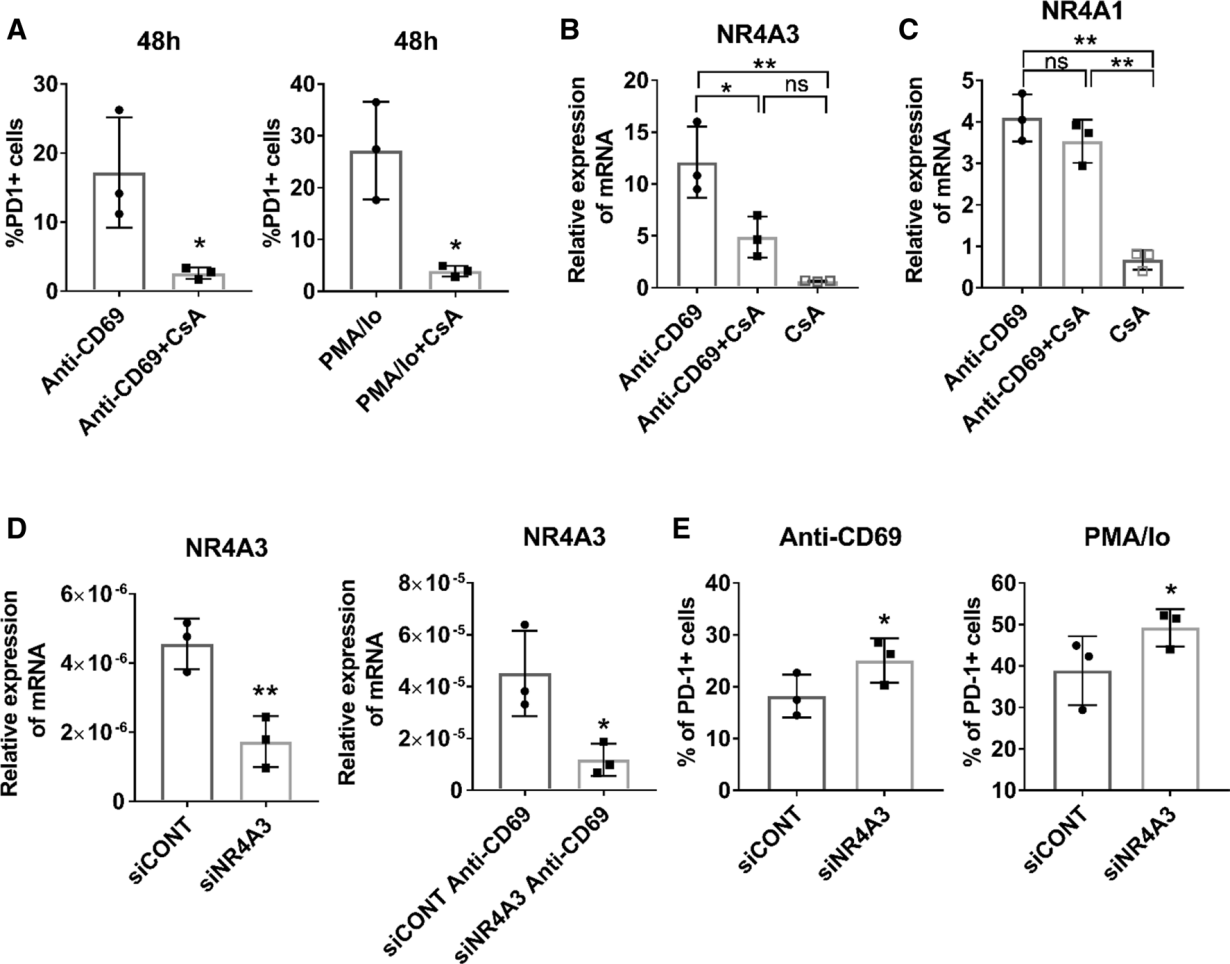
NFAT regulates PD-1 expression induced by CD69 engagement in T cells. **A** Cyclosporine A reduces the expression of PD-1 + in T cells. JKCD69 cells were incubated with crosslinked anti-CD69 with or without cyclosporine A (CsA), or phorbol 12-myristate 13-acetate and ionomycin (PMA/Io) stimulated with or without CsA for 48 h. Data are mean ± SD (*n* = 3), and differences were determined by unpaired *t* test. * *P* < 0.05. **B** Cyclosporine A inhibits CD69-mediated NR4A3 induction. Relative expression of NR4A3 in JKCD69 incubated with anti-CD69 monoclonal antibody, with or without CsA and with CsA in resting cells for 4 h. Data are mean ± SD (*n* = 3), and differences were analysed with one-way ANOVA (Tukey post hoc test). * *P* < 0.05, ** *P* < 0.01. **C** Induction of NR4A1 is independent of NFAT blockade. Relative expression of NR4A1 in JKCD69 with anti-CD69 antibody, with or without CsA and with CsA in resting cells incubated for 4 h. Data are mean ± SD (*n* = 3) and were analysed with one-way ANOVA (Tukey post hoc test). *P < 0.05, **P < 0.01. **D** Relative expression levels of NR4A3 mRNA after siRNA knockdown in resting and anti-CD69 stimulation conditions. Data showed are mean ± SD (*n* = 3), and differences were determined by unpaired *t* test. *P < 0.05. ** *P*<0.01. **E** siRNA interfering of NR4A3 expression enhances PD-1 + cell population. JKCD69 were stimulated with anti-CD69 or PMA/Io for 48 h. Data are mean ± SD (*n* = 3), and differences were determined by paired *t* test. *P < 0.05

CD69 crosslinking triggers a mobilization of Ca^2+^ extracellular influx as well as the activation of NFAT proteins [20–22]. Moreover, NFATc1 is involved in the transcriptional regulation of PD-1 [23]. Thus, we probed whether NFAT activation was responsible for the induction of PD-1 through CD69 binding. Treatment with CsA, which impairs the dephosphorylation of NFAT by calcineurin and its recruitment to the nucleus, prevented the induction of *PD-1* and the *NR4A3* mRNA expression triggered by CD69 activation or the combination of PMA and ionomycin (Fig. 3A, B) [24]. However, CsA did not affect *NR4A1* mRNA expression induced by CD69 (Fig. 3C). In order to determine the contribution of NR4A3 to the regulation of PD-1 expression, we inhibited *NR4A3* expression using a specific siRNA (Fig. 3D). Interestingly, silencing *NR4A3* slightly upregulated PD-1 expression upon induction with both anti-CD69 and PMA/Io stimulation in JKCD69 cells (Fig. 3E).

### PD-1 and CD69 are highly expressed in inflamed human arteries

**Fig. 4:**
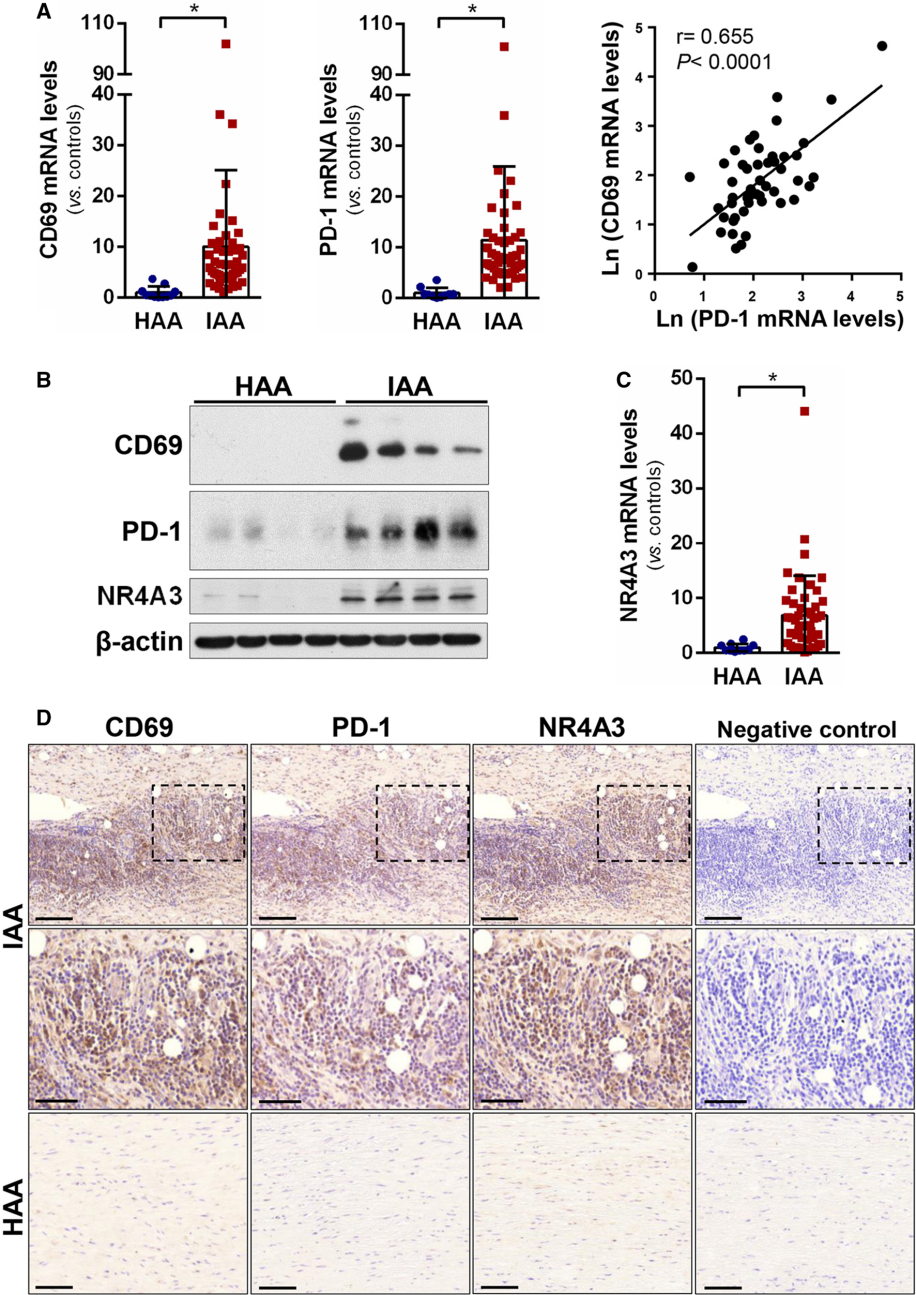
PD-1 and CD69 are highly expressed in inflamed human arteries. **A** PD-1 and CD69 mRNA levels were measured in inflamed abdominal aortic samples (IAA) from patients undergoing open repair surgery for abdominal aortic aneurysm (*n* = 49) and healthy abdominal aortic samples (HAA) from donors (*n* = 11). Data are expressed as mean ± SD *P < 0.05 (Mann–Whitney test). (right panel) Statistically significant positive correlation between CD69 mRNA levels and those corresponding to PD-1 in inflamed aortic samples from patients. The Pearson Product Moment Correlation was applied. **B** CD69, PD-1 and NR4A3 protein levels were evaluated by western blot in aortic lysates from IAA and HAA samples. Representative immunoblots are shown. **C** NR4A3 mRNA levels assessed in the samples described in A). Data are expressed as mean ± SD. *P < 0.05 (Mann–Whitney test). **D** Immunohistochemical analysis of CD69, PD-1 and NR4A3 in haematoxylin counterstained sections from IAA and HAA samples. The indicated areas are shown at high magnification (middle panels). Bar: 100 µm (upper and lower panels) and 50 µm (middle panels). Negative controls in which the primary antibody was omitted are shown on the right

To assess whether CD69 and PD-1 upregulation is associated with chronic vascular inflammatory disease, *CD69*, *PD-1* and *NR4A3* mRNA expression was analysed by RT-PCR in inflamed abdominal aortic samples from aneurysm patients undergoing open repair surgery (*n* = 49) and healthy abdominal aorta from multi-organ donors (*n* = 11). The clinical and demographic data of the patients and donors are collected in Table 2. *CD69* and *PD-1* were highly expressed in inflamed aortas compared with donor samples, and their mRNA levels showed a strong positive correlation (*r* = 0.655 *p* < 0.0001) (Fig. 4A). In addition, CD69 and PD-1 proteins levels were also increased in diseased aortas, as determined by western blotting (Fig. 4B). NR4A3 expression (mRNA and protein) was also upregulated in inflamed aortas, as expected (Fig. 4B, C). Furthermore, immunohistochemical analysis of consecutive aortic sections revealed high CD69 and PD-1 immunostaining in areas enriched in inflammatory cells (Fig. 4D, left and middle panels), and a similar pattern was observed for NR4A3 (Fig. 4D, right panels). PD-1 immunostaining was localized in areas of inflamed arteries enriched in CD3 + cells (Supplementary Fig. 2).

**Table 2:** Clinical features of patients and controls

Clinical parameters	Controls (*n* = 11)	Patients (*n* = 49)
Age (years ± SEM)	61 ± 5.4	71.3 ± 0.66
Males (%)	91	100
Smoking (%)^a^	55	81.6
Hypertension (%)	64	67.3
Diabetes (%)	36	10.2
Hyperlipidemia (%)	27	57.1
Ischaemic cardiomyopathy (%)	0	18.4

*SEM* standard error of the mean

^a^Current and ex-smokers

## Discussion

In this work, we identify a novel TCR-independent induction mechanism of PD-1 through the engagement of CD69 and oxLDL. RNA-Seq analysis of JKwt and JKCD69 cultured with LDL oxidized to a different degree revealed a CD69-dependent and LDL oxidation-dependent response. Cells left untreated or treated with native LDL displayed similar expression profiles, while oxLDL produced differences in gene expression that are more pronounced in JKCD69. These results underscore the specificity of CD69 for oxLDL and identified specific responses triggered by CD69-oxLDL binding. Differences elicited by lipoproteins based on their oxidation degree are in line with previous studies [25–27]. Interestingly, *PD-1* was found to be one of the most overrepresented mRNA in JKCD69 cells treated with oxLDL*.* As expected, oxLDL markedly elevated the expression of NR4A3 and also NR4A1 [9]. In vitro assays confirmed the mRNA and protein induction of PD-1 in JKCD69 in the presence of highly oxLDL. The specificity of the signal and the dependence of NR4A and PD-1 induction through CD69 in T cells was confirmed using an anti-CD69 monoclonal antibody. Interestingly, this finding was corroborated with primary human CD4 + T cells.

PD-1 expression is transiently induced by activating stimuli such as PMA and ionomycin, concanavalin A, anti-CD3/CD28 antibodies or by antigen–TCR engagement and cytokine receptor engagement [28, 29]. Extracellular influx of Ca^2+^ induces the expression of NFAT regulated genes through calcineurin activation, including PD-1 [23]. Our data highlight the role of NFAT as a transcriptional regulator involved in the expression of PD-1 and NR4A3 induced by CD69. However, the expression of NR4A1 was not affected following NFAT inactivation. These results are in accordance with previous studies that evaluated the sensitivity of the NR4A subfamily of nuclear receptors to NFAT pathway inhibitors in mice [30]. However, our data also revealed a slightly increased expression of PD-1 in NR4A3-silenced cells treated with anti-CD69 or with PMA/Io stimulation. Accordingly, recent studies have confirmed a relationship between the expression of NR4A receptors and PD-1 in T cells [31, 32]. Hence, the three NR4A proteins regulate the accessibility of an enhancer located at ~ 23 kb 5′ of the *Pdcd1* transcription start site (TSS) in a number of murine models for exhausted CD8 + T cells [32]. The upregulation of hoxLDL-induced NR4A3 might be functioning as a negative feedback loop to counter-regulate the expression of PD-1 induced by CD69.

The PD-1/PD-L1 pathway contribution in cardiovascular diseases has been thoroughly studied. *Pd-1* and *Ldlr* double knockout mice developed larger atherosclerotic lesions with higher CD4 + and CD8 + T cells and macrophages cell numbers [29]. PD-1 blockade increased atherosclerotic lesion inflammation in hypercholesterolemic *Ldlr*^−/−^ mice, suggesting that PD-1 acts as negative regulator of inflammation in atherosclerosis [29]. Accordingly, cardiovascular complications from immune checkpoint inhibitors therapies as PD-1 and PD-L1 inhibitors are an emerging concern that merits further investigation [33]. Our analysis from inflamed abdominal aortic samples revealed a strong correlation between *PD-1* and *CD69* mRNA expression. Moreover, more than half of the patients analysed had hyperlipidemia. Immunohistochemical analyses revealed strong expression of PD-1, CD69 and NR4A3 in areas enriched in inflammatory cells, in which the regulation of PD-1 through CD69 and NR4A3 might contribute to this chronic vascular inflammatory disease.

The role of CD69 has also been studied in tumour immunology where it has been identified as a negative regulator of the anti-tumour responses. *Cd69*-deficient mice show a reduced tumour growth and the downregulation of CD69 by monoclonal antibodies enhanced the anti-tumour immunity [34, 35]. In addition, CD69 exerted a role in controlling immune inflammatory and autoimmune responses [2, 36]. Conceivably, the regulatory role of CD69 in tumour and inflammatory responses could be mediated, at least in part, by modulation of PD-1 expression.

Our study identifies a new function of CD69 by promoting PD-1 expression in CD4 + T lymphocytes after the engagement with oxLDL that might be responsible for the exacerbated activation state found in the absence of CD69 in the atherosclerosis model. The discovery of this regulation underscores the relevant role of these molecules in inflammatory cardiovascular diseases and provides new knowledge for developing therapeutic strategies.

## Supplementary Information

Below is the link to the electronic supplementary material.

Supplementary file1 (XLSX 189 KB)

Supplementary file2 (PDF 328 KB)

## Data Availability

The data underlying this article are available in the article and in its online Supplementary material.
