# Supplementary material for: CD69-oxLDL ligand engagement induces Programmed Cell Death 1 (PD-1) expression in human CD4 + T lymphocytes
Source: Cell Mol Life Sci. 2022 Aug 5;79(8):468. doi: 10.1007/s00018-022-04481-1 (PMC9355928; doi:10.1007/s00018-022-04481-1)
Supplement: Supplementary file 2 — Supplementary file2 (PDF 328 KB) [file 18_2022_4481_MOESM2_ESM.pdf]

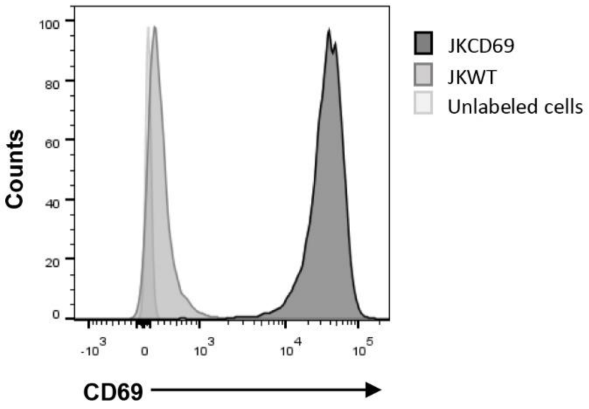

Supplementary Figure 1. CD69 expression levels by flow cytometry. Histograms of the CD69 expression of Jurkat cell line stably transfected or not with CD69 (JKwt and JKCD69, respectively).

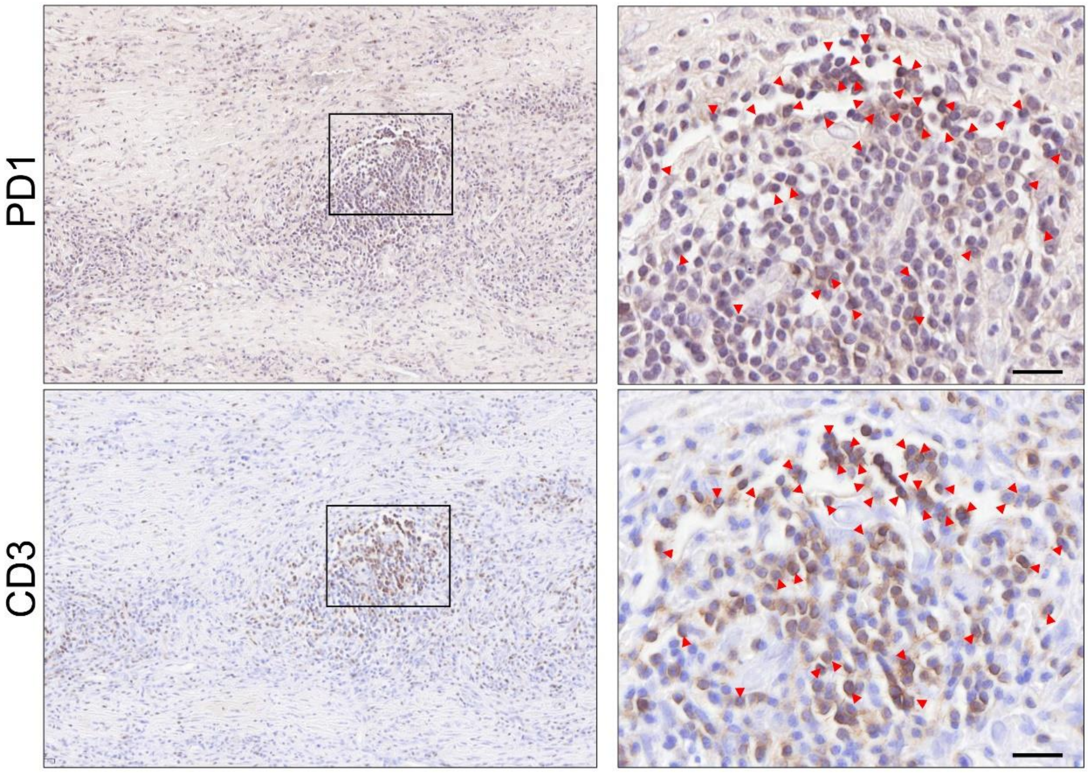

Supplementary Figure 2. **PD-1 is expressed in CD3+ cells in inflamed human arteries**. PD-1 and CD3 immunostaining in consecutive cross-sections corresponding to inflamed abdominal aortic samples (IAA). The boxed areas are shown at a higher magnification on the right and red arrowheads depict cells that express both proteins in consecutive sections. Scale bars: 20 µm.
